# Generation of an 870 kb deletion encompassing the *Skt/Etl4* locus by combination of inter- and intra-chromosomal recombination

**DOI:** 10.1186/s12863-015-0302-0

**Published:** 2015-12-18

**Authors:** Katrin Serth, Anja Beckers, Karin Schuster-Gossler, Maria N. Pavlova, Julia Müller, Mariel C. Paul, Richard Reinhardt, Achim Gossler

**Affiliations:** Institut für Molekularbiologie OE5250, Medizinische Hochschule Hannover, Carl-Neuberg-Str.1, 30625 Hannover, Germany; Max Planck-Genome-Centre Cologne, Carl-von-Linné-Weg 10, D-50829 Köln, Germany; Department of Anaesthesiology and Pain Medicine, University of Washington, Seattle, WA 98001 USA; Gasteiner Str. 31, 10717 Berlin, Germany; Department of Medicine I, Institute of Cancer Research, Medical University of Vienna, Borschkegasse 8a, 1090 Vienna, Austria

**Keywords:** Targeted deletion, Tamere, etl4/skt, Notochord development

## Abstract

**Background:**

Etl4^lacZ^ (Enhancer trap locus 4) and Skt^Gt^ (Sickle tail) are lacZ reporter gene integrations into the same locus on mouse chromosome 2 targeting a gene that is expressed in the notochord of early embryos and in multiple epithelia during later development. Both insertions caused recessive mutations that resulted exclusively in mild defects in the caudal vertebral column. Since notochord-derived signals are essential for formation of the vertebral column the phenotypes suggested that the lacZ insertions interfered with some notochord-dependent aspect of vertebral development. As both insertions occurred in introns it was unclear whether they represent hypomorphic alleles or abolish gene function. Here, we have generated a definitive null allele of the Skt/Etl4 gene and analysed homozygous mutants.

**Results:**

We have introduced loxP sites into three positions of the gene based on additional upstream exons that we identified, and deleted approximately 870 kb of the locus by a combination of inter- and intra-chromosomal Cre-mediated recombinations in the female germ line of mice. This deletion removes about 90 % of the coding region and results in the loss of the SKT/ETL4 protein. Similar to the Etl4^lacZ^ and Skt^Gt^ alleles our deletion mutants are viable and fertile and show only mild defects in caudal vertebrae due to abnormal intervertebral disc development, although with higher penetrance. No other tissue with Skt/Etl4 expression that we analysed showed obvious defects.

**Conclusion:**

The complete loss of Skt/Etl4 function affects only development of caudal notochord derivatives and is compensated for in its other expression domains.

**Electronic supplementary material:**

The online version of this article (doi:10.1186/s12863-015-0302-0) contains supplementary material, which is available to authorized users.

## Background

In higher vertebrates the notochord is a transient rod-like structure in the midline of the embryo. In mouse embryos beginning at embryonic stage E8.5 the notochord arises as a distinct anatomical entity. Its anterior end is close to Radtke’s pouch from which it extends posteriorly to the tip of the tail [1]. Early during development the notochord serves as an essential signalling centre for dorso-ventral patterning of the overlaying neural tube and the formation of the floorplate [2] by secretion of sonic hedgehog (Shh) that activates the hedgehog signalling pathway in adjacent tissues [3]. Likewise, signals from the notochord ventralise the somites [4], segmentally repeated mesodermal units located at either side of the neural tube reviewed in [5], and induce the differentiation of sclerotome cells [6], the precursors of the vertebral bodies, intervertebral discs, neural arches and ribs reviewed in [5]. Consequently, notochord ablation and mutations that affect the formation or maintenance of the notochord lead to malformations of the axial skeleton due to the lack or reduction of sclerotome inducing signals [6].

In addition to inducing sclerotome differentiation and thereby indirectly regulating axial skeleton development notochord cells directly contribute to the vertebral column: at embryonic stage E12 sclerotome cells around the notochord condense in a metameric pattern and mark the future vertebral body and the intervertebral disc (IVD) regions. Concomitantly, cells of the notochord start to expand within the future IVDs and are expelled from the vertebral body regions until they disappear completely [7]. The molecular control of this withering process in the segmentation of the notochord is unknown, but is likely explained by biomechanical forces that squeeze the notochord cells towards the IVD regions [8, 9]. IVDs consist of three main structures: the nucleus pulposus (NP), the annulus fibrosus (AF) and the endplates (EP) reviewed in [10]. Lineage tracing studies revealed that notochord cells persist in the adult spine solely within the NP [11, 12], a gelatinous tissue in the centre of the IVD that produces collagen II and proteoglycans, and serves as a shock-absorbing structure between vertebrae.

Genes that control notochord development in mice have been identified by mutational analyses. The transcription factor FOXA2 as well as the upstream factors TEAD1 and TEAD2 are essential for the initiation of notochord formation [13, 14]. High levels of the transcription factor Brachyury (T) are required for maintenance of the posterior notochord, as haploinsufficiency results in abnormal posterior notochord development and shortened tails [15]. Similarly, the homeodomain transcription factor NOTO is required for normal caudal notochord development and null mutants show interruptions and malformations of the tail axial skeleton [16]. The highly related transcription factors SOX5 and SOX6 regulate extracellular matrix genes and are required for formation of the peri-notochordal sheath, a thick basement membrane surrounding the notochord, that is required for notochord cell survival, and NP development [17].

Also mutations caused by lacZ enhancer and gene trap insertions with reporter gene expression in the notochord led to abnormal vertebral column development. During a screen to identify developmentally regulated genes the enhancer trap line 4 (Etl4^lacZ^) mouse line was established [18]. The integrated lacZ reporter gave rise to expression in the notochord and in the future IVDs as well as in branchial arches, limb buds and embryonic kidney during embryogenesis [19]. The transgene integration produced a recessive mutation characterised by mild tail kinks due to abnormally shaped vertebrae [19]. Similarly, a gene trap insertion termed sickle tail (Skt^Gt^) showed primarily lacZ expression in the notochord, the future IVDs, the mesonephros and in the nuclei pulposi of adult mice [20]. Homozygous Skt^Gt^ mice had tails with caudal kinks due to malformation and mislocation of the NP in the caudal region of the vertebral column [20]. Genomic mapping of the transgene integration sites of Etl4^lacZ^ and Skt^Gt^ showed that both occurred on mouse chromosome 2 within the same genomic locus referred to as the Etl4 or Skt gene [19, 20]. Several transcripts from the Skt/Etl4 locus were described, the longest coding for an approximately 150 kDa protein containing a proline-rich region and a coiled-coil domain with a so far unknown function [20].

Skt/Etl4 expression in the notochord and abnormal development of vertebrae in Etl4^lacZ^ and Skt^Gt^ mutants suggested that the Skt/Etl4 gene affects the function of a gene important for notochord-dependent somite differentiation and subsequent vertebral development. However, since both lacZ insertions occurred within intronic regions of the Skt/Etl4 gene (Etl4^LacZ^ in intron 3, Skt^Gt^ in intron 14 according to Semba et al 2005 [20]) and caused only a fairly mild axial skeleton phenotype, it was unclear whether these insertions completely abolish gene function or represent hypomorphic alleles that affect only some aspect of Skt/Etl4 function in the notochord. Here, we generated a bona fide null allele of Skt/Etl4 to clarify its function during mouse development. Using a combination of gene targeting in ES cells and targeted meiotic recombination [21] we deleted 868 kb of genomic DNA encompassing nearly the whole coding region of Skt/Etl4. Surprisingly, mice homozygous for this deletion were viable and fertile. Despite expression of Skt/Etl4 in multiple tissues during embryonic development the deletion caused only mild malformations of the axial skeleton virtually identical to the Etl4^lacZ^ and Skt^Gt^ alleles.

## Results

### Identification of transcripts derived from the Skt/Etl4 locus

**Fig. 1:**
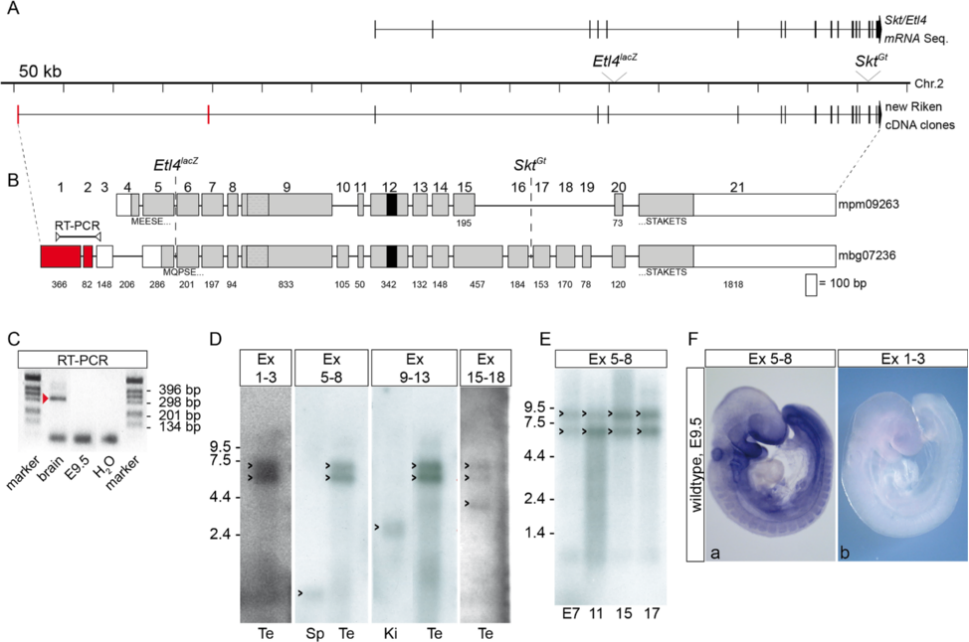
Structure and transcripts of the Skt/Etl4 gene. **a** Scheme of mouse chromosome 2 with the integration sites of Enhancer trap locus 4 (Etl4^lacZ^) and Sickle tail (Skt^Gt^). Above the scheme the distribution of Skt/Etl4 exon sequences obtained from GenBank/NCBI entries NM_178059.5 (Etl4-a), NM_029895.4 (Etl4-b), NM_001081006.1 (Etl4-c), NM_001177630.2 (Etl4-d), NM_001177631.1 (Etl4-e), BC158051.1, BC050016.1, BC026657.1, AB125594.2 (Skt-a), AB125595.2 (Skt-b) is shown, below the exon distribution of Riken cDNA clones mpm09263 and mbg07236. The new exons 1 and 2 located 360 kb and 168 kb upstream of the already published Skt/Etl4 mRNA are indicated in red. **b** Schematic representation of Riken cDNA clones mpm09263 (4573 bp) and mbg07236 (5659 bp). Squares represent exons (*drawn at scale*) with continuous numbering above and sizes in bp below. Above exon 1, 2, 3 the location of the primer pair used in (**c**) is indicated. Grey squares: open reading frame with the respective N- and C-terminal amino acid sequences indicated below. Stippled box: proline-rich region. Black box: coiled-coil region. Red box: new exons 1 and 2. **c** RT-PCR with primers located in exon 1 and 3 (depicted in (**b**)) with poly (A^+^) RNA isolated from adult brain and embryo stage E9.5. Negative control (water) contains no cDNA. Red arrowhead marks PCR product used for sub-cloning and sequencing. **d** Northern blots with adult testis (Te), spleen (Sp) and kidney (Ki) poly (A^+^) RNA hybridized with Skt/Etl4 probes specific for exons indicated above. Detected transcripts of around 7, 6, 4, 3 and 1 kb in size are indicated by black arrowheads. **e** Northern blot with poly (A^+^) RNA from various embryo stages (indicated below) hybridized with exon 5-8 specific probe shows approx. 8 and 7 kb transcripts (*arrowheads*). **f** Whole mount in situ hybridization of wild type stage E9.5 embryos (**a**, **b**) with Skt/Etl4 exon-specific probes indicated at the top

To identify the gene detected by the Etl4^lacZ^ insertion we isolated and sequenced overlapping BAC and PAC clones of the genomic Etl4 region on mouse chromosome 2 (prior to publication of the mouse genome sequence and identification of the Skt gene). Within this genomic region we identified two cDNA clones, mpm09263 and mbg07236, from the Kazusa Mouse cDNA Project [www.kazusa.or.jp/rouge [22], which contained exons flanking both, the Etl4^lacZ^ and the Skt^Gt^ insertions identified by Semba et al [20]. Both cDNA clones extensively overlap with other Skt/Etl4 mRNA sequences (Fig. 1a), and contain two additional so far unknown exons (red lines in Fig. 1a) located around 360 kb and 168 kb upstream of the thus far known most 5’exon in the genomic region. These cDNA clones contained 21 exons that are distributed over approximately 871 kb of genomic sequence (Fig. 1a), and extend the genomic region of the Skt/Etl4 gene listed in GenBank entries at NCBI (Fig. 1a). The longest open reading frame starts in exon 4 and ends in exon 21 (Fig. 1b). According to this gene structure the Etl4^lacZ^ and the Skt^Gt^ insertions occurred in intron 4 and 15 of the gene, respectively.

To verify that the newly identified exons 1 and 2 are indeed part of the Skt/Etl4 gene we analysed poly (A^+^) RNA isolated from adult brain and stage E9.5 embryos by RT-PCR using primers pair located in exon 1 and 3 (Fig. 1b). We obtained the expected PCR-Fragment (validated by sequencing, data not shown) from adult brain mRNA (Fig. 1c lane 1), but not from E9.5 mRNA (Fig. 1c lane 2). Furthermore a probe encompassing exon 1–3 hybridized to the same transcripts of around 7 and 6 kb as probes specific for exons 5–8, exons 9–13 and exons 15–18 (Fig. 1d) in Northern blot hybridizations of poly (A^+^) RNA from adult testis, supporting that the newly identified exons contained in mbg07236 are part of the Skt/Etl4 gene. In addition to the 7 and 6 kb transcripts detected in testis we observed an approximately 1 kb transcript in spleen with the exon 5–8 probe (Fig. 1d, Sp), an approx. 3 kb transcript in kidney with the exon 9–13 probe (Fig. 1d, Ki) and an approx. 4 kb transcript in testis with the exon 15–18 probe (Fig. 1d, Te), suggesting the generation of complex tissue-specific transcripts potentially derived from different promoters. In mRNAs from various embryonic stages we detected two transcripts of around 8 kb and 6 kb (Fig. 1e) with the exon 5–8 (Fig. 1e) but not with the exon 1-3 probe (not shown), suggesting that exon 1–3 are specific for testis (and brain as detected by RT-PCR) expressed transcripts of Skt/Etl4. Consistent with the Northern Blot results whole-mount in situ hybridizations (WISH) on E9.5 embryos with the exon 5-8 (*n* = 30) but not with the exon 1–3 (*n* = 10) probe detected expression in E9.5 embryos (Fig. 1f a, b).

### Expression of Skt/Etl4 mRNA

**Fig. 2:**
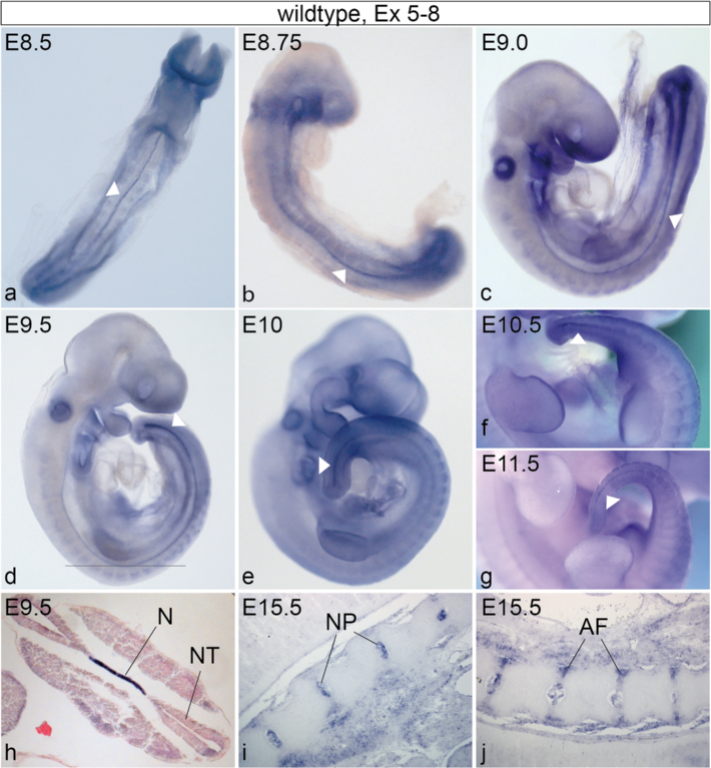
Skt/Etl4 mRNA expression in early embryos. **a**-**g** Whole mount in situ hybridizations on wt E8.5 to E11.5 embryos with an exon 5-8 specific probe showing graded notochord expression (E8.5–8.75:*n* = 11; E9.0: *n* = 5; E9.5: *n* = 30; E10: *n* = 3; E10.5: *n* = 3; E11.5: *n* = 5). Black line in **d** indicates the position of a transverse HE stained section of the embryo shown in **h**. **i, j** Section in situ hybridization of wt E15.5 embryos (*n* = 3) showing expression of Skt/Etl4 within the developing nucleus pulposus (NP, **i**) and annulus fibrosus (AF, **j**)

To get a comprehensive picture of the expression pattern and to detect expression domains that might have been missed in previous experiments using the lacZ reporter gene in Etl4^lacZ^ or Skt^Gt^ mice [19, 20] we performed in situ hybridizations with a Skt/Etl4 specific probe. Consistent with the β-galactosidase staining pattern of the lacZ alleles [19, 20] we found expression in the notochord at similar levels along its entire length by WISH of E8.5 to E8.75 embryos (Fig. 2a, b). However, beginning at E8.75 up to approximately E11.5 Skt/Etl4 expression in the notochord appeared graded with high expression in the caudal region (Fig. 2c-g). Furthermore we detected Skt/Etl4 expression within the otic placode (Fig. 2c), the branchial arches (Fig. 2c) and the AER of the fore- and hindlimbs (Fig. 2e, f).

**Fig. 3:**
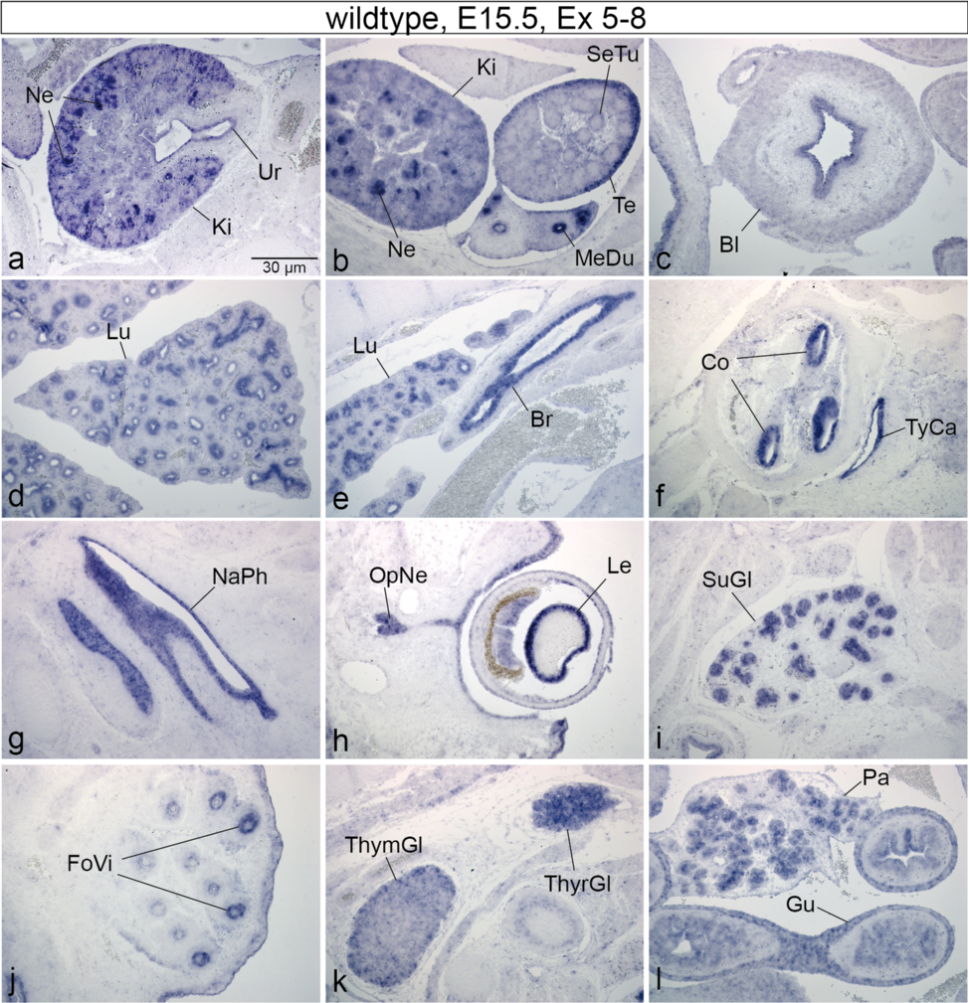
Skt/Etl4 expression during organogenesis. **a**-**l** In situ hybridizations of sagittal sections of wt E15.5 embryos with an exon 5–8 specific probe showing expression in developing nephrons (Ne, **a**) the cortical region of kidney (Ki, **a**, **b**), ureter epithelium (Ur, **a**), mesonephric duct (MeDu, **b**) bladder epithelium (Bl, **c**), cortical region of testis (Te, **b**) and seminiferous tubules (SeTu, **b**), lung epithelium (Lu, **d**, **e**), bronchus (Br, **e**), cochlea epithelium (Co, **f**), tympanic cavity epithelium (TyCa, **f**), nasopharynx epithelium (NaPh, **g**), lens (Le, **h**), optic nerve (OpNe, **h**), primordia of follicles of vibrissae (FoVi, **j**), ducts of submandibular gland (SuGl, **i**), thymus gland (ThymGl, **k**), thyroid gland (ThyrGl, **k**), exocrine acini of pancreatic primordium (Pa, **l**), gut epithelium and wall (Gu, **l**)

In order to identify additional tissues expressing Skt/Etl4, we performed section in situ hybridizations (SISH) with a Skt/Etl4 specific probe on transverse sections of stage E15.5 wt embryos (*n* = 3) when most of the vital organs are present or developing. In E15.5 embryos when notochord cells are incorporated into the intervertebral discs Skt/Etl4 expression persisted in the developing NP and AF throughout the whole vertebral column (Fig. 2i and j, [20]). In addition to the expression in the emerging IVDs we verified the expression in the kidney (Ki, Fig. 3a, b) with strong signals within the cortical region presumably in developing nephrons (Ne, Fig. 3a, b). Also the epithelium of the ureter (Ur, Fig. 3a), the mesonephric duct (MeDu, Fig. 3b) and the bladder (Bl, Fig. 3c) were positive for Skt/Etl4. In testis expression was strongest within the cortical region (Te, Fig. 3b) and less within the outer region of the seminiferous tubules (SeTu, Fig. 3b). We additionally identified tissues were Skt/Etl4 expression was not known hitherto: the epithelium of the lung (Lu, Fig. 3d, e) the bronchus (Br, Fig. 3e), as well as the epithelium of the cochlea (Co, Fig. 3f), the tympanic cavity (TyCa, Fig. 3f) and the nasopharynx (NaPh, Fig. 3g). In the developing eye we found that in addition to the lens (Le, Fig. 3h, [20] and inner layer of the retina [20], Skt/Etl4 also is expressed within the optic nerve (OpNe, Fig. 3h). The primordia of follicles of vibrissae associated with the upper lip (FoVi, Fig. 3j) as well as the ducts of the submandibular gland (SuGl, Fig. 3i), the developing thymus gland (ThymGl, Fig. 3k) and thyroid gland (ThyrGl, Fig. 3k) display Skt/Etl4 expression. Expression was also detected within the pancreatic primordium most likely within the exocrine acini (Pa, Fig. 3l) and the wall and epithelium of the gut (Gu, Fig. 3l).

### Targeted insertion of lacZ reporter genes into Skt/Etl4 exons 1 and 5

In both mouse lines, Etl4^lacZ^ and Skt^Gt^, the insertion of a lacZ transgene resulted in similar very mild phenotypic changes [19, 20]. Both integrations occurred within introns (Fig. 1a) and in the Skt^Gt^ allele a truncated protein of 998 aa fused to β-galactosidase could potentially be generated from the mutated locus [20], raising the possibility that these mutations represent hypomorphic alleles. To evaluate the phenotype of the complete loss of the gene we set out to generate a bona fide null allele of Skt/Etl4.

The presence of various transcripts presumably expressed from different promoters [Fig. 1 d and e; and 20], and the large genomic distance between many coding exons (Fig. 1a) precluded a simple targeting strategy to ensure the complete elimination of Skt/Etl4 function. Therefore, we decided to introduce lacZ reporter genes with strong transcriptional termination signals (triple poly (A) [23] and loxP sites into two different regions of the Skt/Etl4 locus that are parts of differentially expressed transcripts. The termination signal should prevent the expression of further downstream sequences from a given promoter, the loxP sites should allow us to delete portions of the gene by site directed recombination. Moreover the integration of the lacZ reporter into different exons should allow us to readily examine the transcriptional activity of the gene from different transcriptional start sites.

**Fig. 4:**
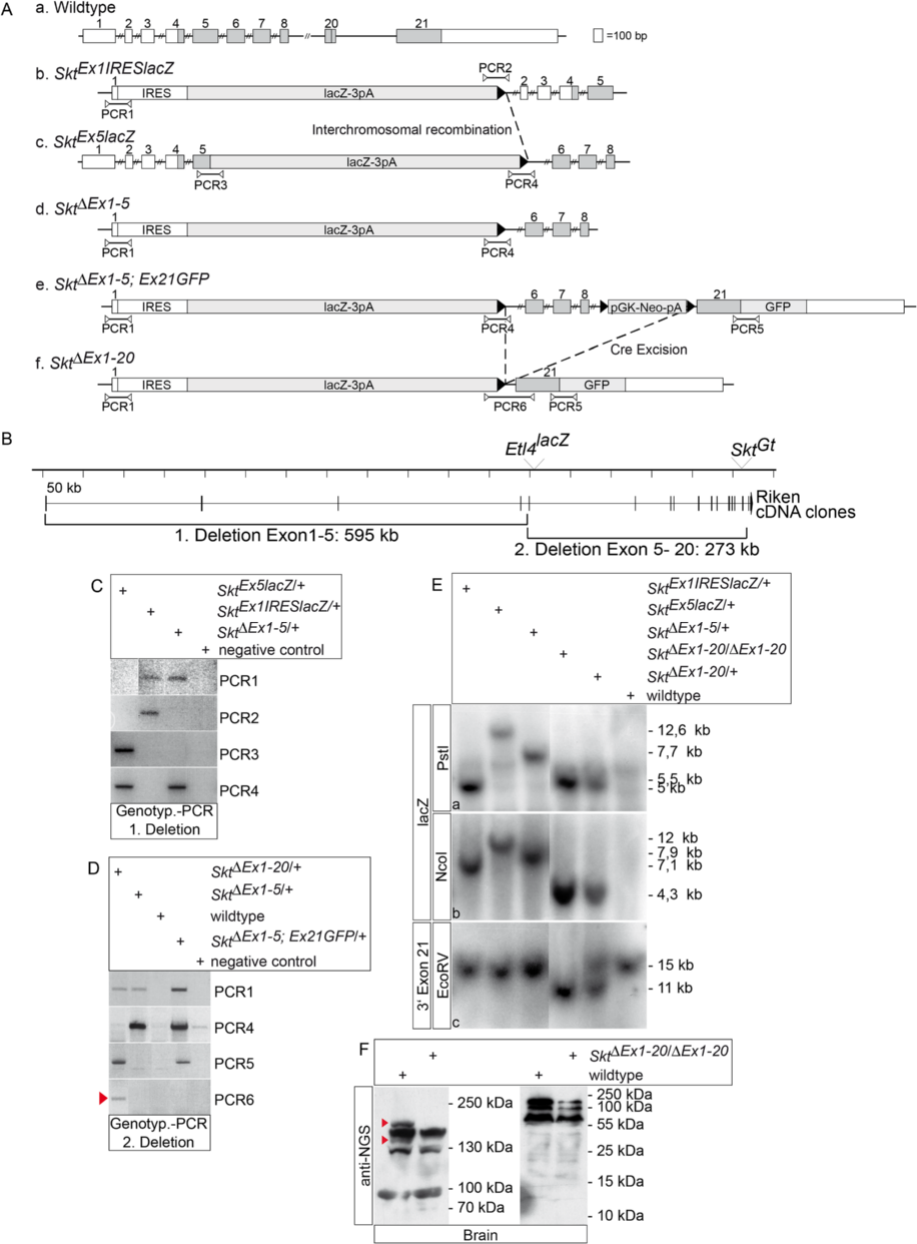
Skt/Etl4 gene inactivation strategies. **a** Overview of Skt/Etl4 wt (*a*) and mutant alleles (*b*-*f*) that were generated by gene targeting. (*b*) Integration of an IRES-lacZ-triple poly **a** into exon 1. Floxed PGK-Neo-pA was removed with Cre recombinase leaving a single loxP site 3’to lacZ. (*c*) LacZ-triple poly **a** integration into exon 5 with 3’loxP site after Cre removal of the floxed PGK-Neo cassette. (*d*) Cre-induced TAMERE between loxP sites of Skt^Ex1IRESlacZ^ (*b*) and Skt^Ex5lacZ^ (*c*) alleles in the mouse resulting in 595 kb genomic deletion between exon 1 and 5 (depicted in B). (*e*) Integration of floxed PGK-Neo cassette together with a GFP-tagged exon 21into the Skt^ΔEx1-5^ allele. (*f*) Cre mediated excision of 273 kb genomic sequences between integrated loxP sites in the Skt^ΔEx1-5, Ex21GFP^ allele (depicted in B). Numbered boxes represent Skt/Etl4 exons, coding exons are marked in grey. lacZ-3pA: β-Galactosidase gene with triple poly **a** signal. Black triangle: lox P site. IRES: internal ribosome entry site. PGK-Neo-pA: Neomycin selection cassette with PGK promoter and poly **a** signal. GFP: green fluorescence protein tag with stop signal. PCR1 to PCR6 shows the positions of Primer pairs that were used for mouse genotyping shown in (**c**) and (**d**). **b** Scheme of the Skt/Etl4 locus drawn to scale showing the two deletion steps to eliminate Skt/Etl4 function. First TAMERE between the Skt^Ex1IRESlacZ^ and Skt^Ex5lacZ^ alleles generated a 595 kb deletion between exon 1 and 5. Second around 273 kb genomic DNA between exon 5 and 21 were deleted by Cre-mediated intrachromosomal excision. **c** PCR Genotyping of offspring after the first deletion event between exon 1 and 5 (see B) with Primer pair combinations PCR 1 to 4 depicted in **a** from breedings of wt males with Skt^Ex1IRESlacZ/Ex5lacZ^, ZP3::Cre females. Mice carrying the 595 kb deletion (Skt^ΔEx1-5^ allele, see A d) were positive for PCR 1 and 4 and negative for PCR 2 and 3. **d** PCR Genotyping of offspring’s after the second deletion event between exon 5 and exon 21 (see B) with PCR Primer pair combinations PCR1, 4, 5 and 6 depicted in **a** from breeding’s of wt males with Skt^ΔEx1-5; Ex21GFP^, ZP3::Cre females. Only when 273 kb of genomic DNA was removed positive signals with PCR 1 and 5 were obtained. In addition PCR 6 specifically detects the whole 870 kb deletion due to the flanking primer positions (red triangle). **e** Verification of chromosomal rearrangements by Southern blot analysis with genomic mouse DNA digested with PstI (**a**) or NcoI (**b**) and hybridized with a lacZ specific probe for discrimination of the different Skt/Etl4 alleles that were generated. A polymorphism obtained with EcoRV-digested genomic DNA (**c**) hybridized with a probe downstream of exon 21 (3‘exon21) probe was used to demonstrate the presence of the wt, heterozygous or homozygous Skt^ΔEx1-20^ alleles in mice. The corresponding restriction maps with the expected fragment sizes of Skt/Elt4 wt and mutant alleles are shown in Additional file 3: Figure S3. **f** Detection of endogenous SKT/ETL4 protein by Western blot analysis using anti-NGS antibody and protein lysates isolated from adult brain of wt and homozygous Skt^ΔEx1-20^ animals. Red arrowheads point to bands missing in lysates of mutant tissue

We introduced an IRES driven lacZ reporter gene fused to triple poly (A) into the most 5’exon (Skt^Ex1IRESLacZ^ allele, Fig. 4a b) to disrupt the transcript specifically detected in brain and testis (Fig. 1c, d). Likewise we introduced a lacZ triple poly (A) cassette into exon 5 (Skt^Ex5LacZ^ allele, Fig. 4a c), to disrupt transcripts expressed during embryogenesis. Correctly targeted ES cells were used to establish mouse lines carrying the Skt^Ex1IRESLacZ^ and the Skt^Ex5LacZ^ alleles (Fig. 4f, and data not shown). The presence of exon 1 containing transcripts was analysed by lacZ staining of embryos and adult tissues of mice carrying the Skt^Ex1IRESLacZ^ allele. Consistent with RT-PCR and WISH results (Fig. 1c and f) we found no lacZ activity during embryogenesis (data not shown). LacZ staining of adult Skt^Ex1IRESLacZ^ organs (brain, testis, epididymis, skeletal muscle, spleen, heart, salivary gland, small intestine, stomach, kidney, liver, lung) revealed only specific lacZ staining in the testis (*n* = 2) and the epididymis (*n* = 2; Additional file 1: Figure S1 A, and data not shown). The staining results confirm the Northern blot results that we obtained with the exon 1-3 probe (Fig. 1d) but are at odds with the RT-PCR results obtained with brain mRNA, where exon 1 potentially is expressed at low levels. Homozygous mice carrying the insertion of the lacZ-stop cassette into exon1 were viable and fertile and had no obvious skeletal phenotypes (data not shown) indicating that disruption of Skt/Etl4 in its 5’most exon did not affect the function during vertebrae development.

With the Skt^Ex5LacZ^ allele we observed specific lacZ reporter gene expression in stage E10.5 (*n* = 6) and E11.5 (*n* = 10) embryos in the notochord (white triangles in Additional file 1: Figure S1 B b, c, e and f), the optic vesicle, the otic placode, the eye, the AER of the fore- and hindlimbs and on the surface of the branchial arches (Additional file 1: Figure S1 B b, c, e, f). This expression pattern was nearly identical to endogenous Skt/Etl4 expression detected with the exon 5 to 8 probe in whole mount in situ hybridizations (Fig. 2e-g) and similar to Etl4^lacZ^ embryos of the same age [19]. Similar to Skt^Ex1IRESLacZ^ mice homozygous Skt^Ex5lacZ^ mice were viable and fertile without obvious external phenotype (data not shown). To test if the termination signal 3’to the lacZ insertion in exon 5 prevents transcription of downstream exons we analysed the presence of exon sequences upstream and downstream of exon 5 in Skt/Etl4 mRNA by RT-PCR using RNA from wt, heterozygous and homozygous Skt^Ex5LacZ^ mutant E10.5 embryos (for primer positions see Additional file 2: Figure S2A). As expected exons 4 and 5 upstream of lacZ were detected in wt and mutant embryos (PCR1, Additional file 2: Figure S2 B), as well as a Skt/Etl4-lacZ fusion transcript in embryos containing the Skt^Ex5LacZ^ allele (PCR2, Additional file 2: Figure S2 B). RT-PCR with primers binding to exon 4 and 7 detected a fragment in which exon 4 was fused to exon 6 revealing an alternative splicing event, which removes the targeted exon 5 (PCR3, Additional file 2: Figure S2 B). In addition, further downstream sequences were still expressed in mutant embryos (PCR4, Additional file 2: Figure S2 B). Removal of exon 5 causes an in-frame deletion of 96 of the total 1373 amino acids. Thus, most likely, this allele does not represent a null allele and therefore was not analysed further.

### Deletion of the whole locus to eliminate Skt/Etl4 gene function

Since the Skt^Ex5LacZ^ allele did not prevent the generation of Skt/Etl4 transcripts that can give rise to likely functional protein (s) we set out to remove the vast majority of the coding region using a two step deletion strategy based on the loxP sites present in the targeted alleles and an additional targeting event into exon 21 (outlined in Fig. 4a, b).

During the generation of the Skt^Ex1IRESLacZ^ and Skt^Ex5LacZ^ alleles a floxed PGK-Neo cassette was introduced in both cases 3’to the lacZ reporter. After removal of the neo gene by Cre-mediated recombination a single loxP site (black triangle in Fig. 4a b, c) in the same orientation remained in the Skt^Ex1IRESLacZ^ and Skt^Ex5LacZ^ alleles. We used these loxP sites in combination with Cre expression during oogenesis to delete 595 kb of genomic DNA between exon 1 and exon 5 by targeted meiotic inter-chromosomal recombination [TAMERE 21]. Heteroallelic Skt^Ex1IRESLacZ^/^Ex5LacZ^ females carrying a ZP3::Cre transgene were mated with wild type males and offspring analysed for the presence of the inter-chromosomal recombination event (Fig. 4a d Skt^ΔEx1-5^ allele, and 4b). Among the first 20 offspring we identified a female with the desired deletion by PCR analyses using four different primer pairs that amplified DNA fragments specific for the various alleles (Fig. 4c, Primer pair position in Fig. 4a). Successful inter-chromosomal recombination was indicated by PCR products obtained with PCR 1 and 4 and lack of products with PCR 2 and 3, which differentiates between the deletion and the presence of the initial Skt^Ex1IRESLacZ^ and Skt^Ex5LacZ^ alleles (Fig. 4c). The correct recombination event that removes the first 4 exons of the gene was validated by Southern blot analysis, which showed the expected polymorphisms between the three alleles (Fig. 4e). Mice homozygous for the 595 kb Skt^ΔEx1-5^ deletion were viable and fertile and did not show any obvious phenotype (data not shown), indicating that the portions of Skt/Etl4 essential for vertebral development were still functional.

To delete the major part of the coding sequence contained in further 273 kb of genomic DNA we generated ES cells from homozygous Skt^ΔEx1-5^ blastocysts, targeted exon 21 by introducing a floxed PGK-Neo cassette (Skt^ΔEx1-5; Ex21GFP^ allele, Fig. 4a e), and generated mice carrying this allele. Intrachromosomal recombination (deletion of exons 5–20) was achieved in female mice harbouring the Skt^ΔEx1-5; Ex21GFP^ allele together with the ZP3::Cre transgene. Offspring carrying the deletion were identified by PCR analyses with 4 different primer pair combinations (Fig. 4d, Primer pair positions in Fig. 4a). Only mice with the second genomic deletion of 273 kb between exon 5 and 21 (Fig. 4b) generate PCR products with PCR1, 5 and 6 in combination with lack of a product with PCR4, which distinguishes the second deletion and the initial Skt^ΔEx1-5^ and Skt^ΔEx1-5; Ex21GFP^ alleles (Skt^ΔEx1-5^ allele, Fig. 4a f and d). Southern blot analysis using a lacZ specific and a genomic probe located downstream of exon 21 (3’Exon21) and three different restriction digests of genomic mouse DNA (Fig. 4e and Additional file 3: Figure S3) confirmed successful deletion of exons 1 to 20 of Skt/Etl4. Despite the deletion of about 88 % of the N-terminal portion of the protein homozygous Skt^ΔEx1-20^ mice were viable and fertile.

To test whether the truncated C-terminal portion of the protein comprising 167 AA encoded by exon 21 is generated we performed Western blot analysis with a polyclonal SKT/ETL4-specific antibody directed against a C-terminal peptide (anti-NGS, see Materials and Methods), which detects SKT/ETL4 protein expressed in CHO cells with the expected size of approximately 150 kDa (data not shown). In protein lysates of adult wild type brain we detected in addition to several other cross-reacting proteins a 200 kDa and a 150 kDa protein species, which were not present in lysates of homozygous Skt^ΔEx1-20^ mice (Fig. 4f, red triangles in the left panel) and likely represent two variants of the SKT/ETL4 protein. We did not see any truncated version of the SKT/ETL4 protein at the predicted approximate size of 16,4 kDa in brain lysates of homozygous Skt^ΔEx1-20^ mice in a Western blot after separation of proteins by SDS-PAGE in higher percentage gels (Fig. 4f, right panel), indicating that no truncated SKT/ETL4 protein is present in these mice. Thus, the established the Skt^ΔEx1-20^ mouse line carrying a 868 kb deletion that removes about 90 % of the N-terminal coding sequence should represent a bona fide null allele of the Skt/Etl4 gene.

### Sickle tail null mice display malformations of caudal IVDs

**Fig. 5:**
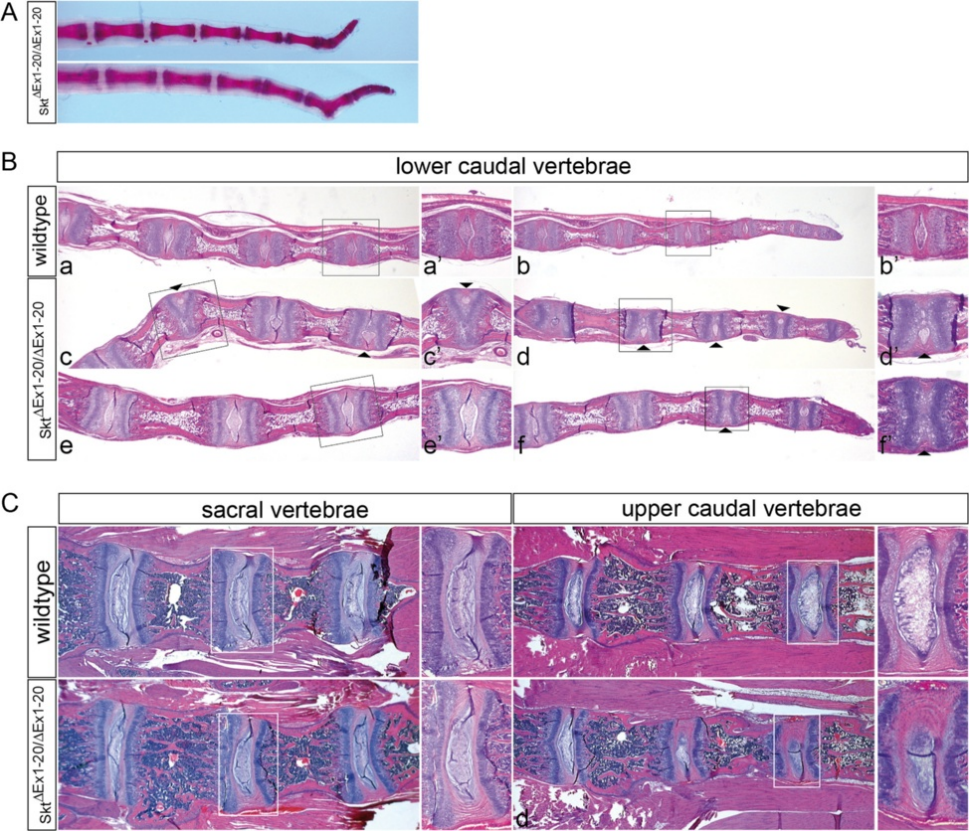
Skt/Etl4 deletion mice display defects in caudal IVDs. **a** Skeletal preparation of tails from 4-week old homozygous Skt^ΔEx1-20^ mice. **b** Haematoxylin-Eosin (HE) stained sagittal sections of tails from the lower caudal region of 3-week old wt (*a-b*) and two homozygous Skt^ΔEx1-20 2^ mice (*c-f*). Black arrowheads in (*c* and *c*‘, *d* and *d*‘) point to shifted, in (*f* and *f*‘) to absent NP. *a*‘-*f*‘show higher magnifications of the regions marked in (*a*-*f*). **c** HE stained frontal sections of the vertebral column from the sacral and upper caudal region of 3-week old wt (*a*-*b*) and homozygous Skt^ΔEx1-20^ (*c*-*d*) mice. (*a*‘-*d*) ‘show higher magnifications of regions marked in (**a**-**d**)

External observation (at least *n* = 12) and skeletal preparations (*n* = 3) revealed that most of the adult homozygous mutant animals displayed kinks within the tails (Fig. 5a) similar to the phenotype described for Etl4^lacZ^ [19] and Skt^Gt^ [20] mice. For histological analysis paraffin sections of tails from 3-week old wt (*n* = 4) and homozygous Skt/Etl4 mutant mice (*n* = 6) were stained with Haematoxylin-Eosin (HE, Fig. 5b). We found aberrations in the morphology of the intervertebral discs (IVDs) that arose mostly in caudal vertebrae as shown in two examples of Skt^ΔEx1-20^ tails (Fig. 5b c-f). Normally in wt mice the NP of the IVDs is centrally located (Fig. 5b a and a’, b and b’), which we also observed in IVDs of Skt^ΔEx1-20^ mutants (Fig. 5b e and e’). However, in several Skt^ΔEx1-20^ mutant IVDs the NP was shifted to the periphery (arrowheads Fig. 5b c, d and higher magnification in c’, d’) or in rare cases not present at all (arrowhead in Fig. 5 b f and higher magnification in f’) in homozygous Skt^ΔEx1-20^ mutants. In addition the fibrous layer of the AF surrounding the NP was reduced in size or not present at all (Fig. 5b c’) resulting in some cases in the direct contact of adjacent vertebral bodies. In more anterior IVDs (Fig. 5c, upper caudal vertebrae) we found only a slight lateral shift of the nucleus pulposus in one IVD of one of four analysed mutant animals (Fig. 5c d and higher magnification in d’) but no other abnormalities compared to wt (*n* = 2). In IVDs of the sacral region no structural alterations of the NP or AF in homozygous mutant (*n* = 4) mice were detected (Fig. 5c, sacral vertebrae). Together our results highly correspond with the phenotype described for the Etl4^lacZ^ and Skt^Gt^ mutants, where only defects in size and position of the NP and AF, and corresponding vertebral malformations were observed caudally, but not in other regions of the vertebral column [19, 20].

### Skt/Etl4 null mice do not display major defects in the notochord

**Fig. 6:**
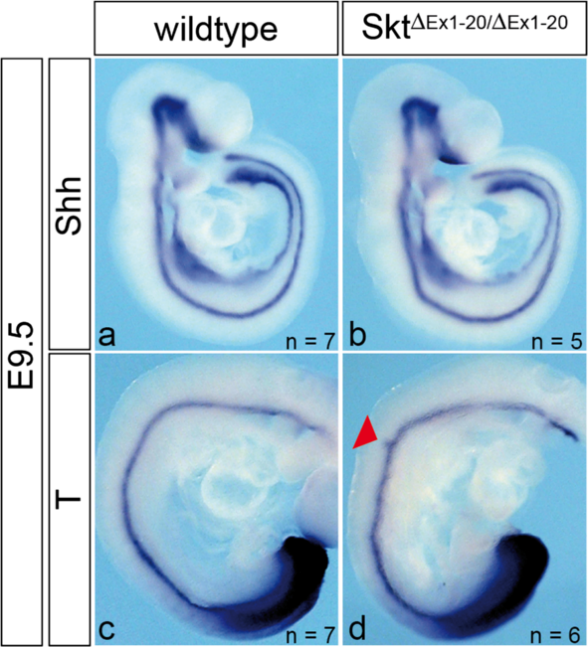
Notochord marker expression in Skt/Etl4 deletion embryos. Whole mount in situ hybridization of E9.5 wt and Skt/Etl4 mutant embryos with the notochord marker sonic hedgehog (Shh, **a**, **b**) and Brachyury (T, **c**, **d**). Red arrowhead point to a T expression abnormality seen in one out of six embryos that were analysed

During mouse embryogenesis between E12 and E13 notochordal cells in the region of the future disc transform and form the later NP and AF. The appearance of the previously described abnormalities in caudal IVDs of Skt/Etl4 deletion mutants may arise from defects in the formation or differentiation of the notochord. To analyse if the Skt/Etl4 deletion leads to obvious notochord defects early during development we hybridized stage E9.5 embryos with probes for the notochordal markers Sonic hedgehog [Shh 3] and Brachyury [T 24]. We observed no differences in the expression pattern of Shh in wt (*n* = 7) and in homozygous Skt/Etl4 (*n* = 5) deletion mutant embryos (Fig. 6a, b). Likewise expression of T at embryonic stage E9.5 was indistinguishable between wt (*n* = 7) and Skt/Etl4 (*n* = 6) mutants with the exception of an ectopic expression domain at the forelimb level in one out of six analysed mutants (red arrowhead Fig. 6d) indicating that the loss of Skt/Etl4 expression does not have a major impact on early notochord development.

### Histological analysis of adult Skt/Etl4 mutants

**Fig. 7:**
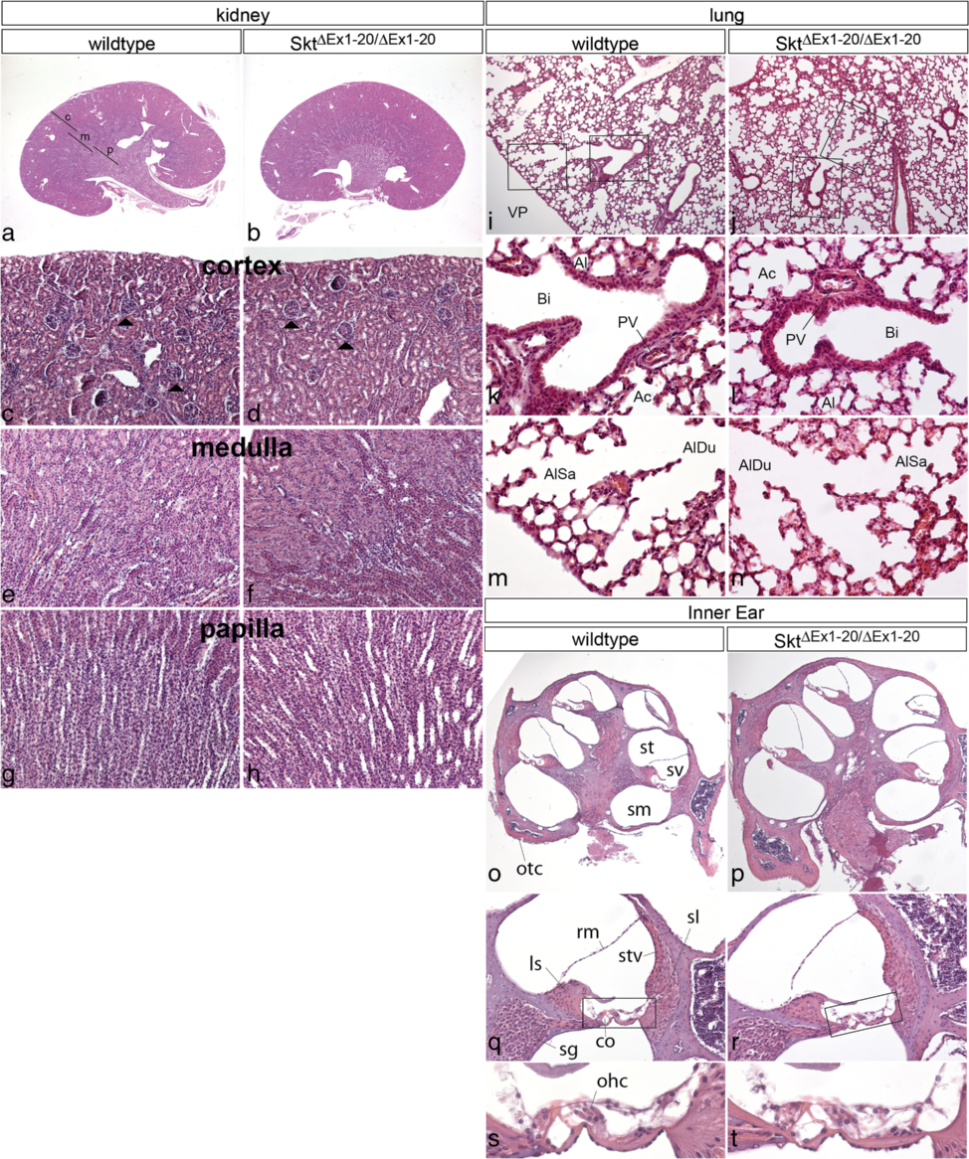
Histological analysis of kidneys, lungs and inner ears of Skt^ΔEx1-20^ mice. HE staining of organs isolated from 2 week old wt and Skt^ΔEx1-20^ mice. **a**-**b** overviews of longitudinal sections through the kidney and magnifications of the cortex (**c**), medulla (**m**) and papilla (**p**) regions **c**-**h** indicated by lines in **a**. Arrowheads in **c** and **d** point to the cortex-specific Glomerular capsules. **i**-**j** Sections through lung tissue. Rectangles in **i** and **j** indicate the regions shown at higher magnification in **k**-**n**. Al: Alveolus, Bi: Bronchiole, PV: pulmonary vessel, Ac: Acini, AlSa: alveolar sac, AlDu: alveolar duct. **o**-**r** Midmodiolar cochlea sections. Rectangles in **q** and **r** indicate the regions shown at higher magnification in **s** and **t**. st: scala tympani, sv: scala vestibuli, sm: scala media, otc: otic capsule, ls: spiral limbus; rm: Reissner‘s membrane, stv: stria vascularis, sl: spiral ligament, co: organ of corti, sg: spiral ganglion, ohc: outer hair cells

As described earlier Skt/Etl4 is a gene expressed in multiple tissues during embryogenesis. Therefore, we analysed various tissues of juvenile Skt/Etl4 deletion mutants for histologically detectable abnormalities. In HE stained longitudinal kidney sections we found no obvious differences in the overall appearance (Fig. 7b) or structure of the cortex (Fig. 7 c, d), the medulla (Fig. 7 e, f) and papilla (Fig. 7 g, h), or the number of glomeruli (data not shown) between wild type (*n* = 2) and mutants (*n* = 2). Other organs that exhibited a thus far not described expression of Skt/Etl4 were epithelia of the lung and the cochlea. We analysed lung tissue of 2 week old wt (*n* = 3) and homozygous Skt^ΔEx1-20^ (*n* = 2) mice with HE staining and could not detect any obvious difference in the tissue structure between both phenotypes (compare Fig. 7i, k, m with j, l, n). Likewise cochleae from the same stage did not exhibit any obvious variations between wt (*n* = 2) and homozygous Skt^ΔEx1-20^ mice (*n* = 2; compare Fig. 7o, q, s with p, r, t).

## Discussion

The Skt/Etl4 gene was independently identified by two insertions of lacZ reporter constructs (Etl4^lacZ^ and Skt^Gt^) into different introns of the locus [19, 20]. Both alleles represent recessive mutations causing mild defects that were restricted to the caudal axial skeleton although Skt/Etl4 is expressed in the notochord along its entire length as well as in numerous other tissues, raising the question whether Etl4^lacZ^ and Skt^Gt^ represent hypomorphic Skt/Etl4 alleles. To conclusively address the significance of Skt/Etl4 for development we analysed the gene structure and transcripts, consecutively deleted most of the coding region and characterised this null allele.

Our analyses identified additional exons 5’to the published gene structure extending the gene by 576 kb and indicating that Skt/Etl4 spans at least 871 kb. Consistent with previous observations [20] we found two major transcripts of approximately 8 and 6 kb in embryos of various stages. We identified additional tissue-specific transcripts of different sizes, the newly identified exons being only present in transcripts in testis and brain, which underscores the complex regulation likely involving different promoters in addition to differential splicing noted by Semba et al. [20]. The use of different promoters is further supported by the differential expression of lacZ from the Skt^Ex1IRESLacZ^ and the Skt^Ex5lacZ^ alleles. Since lacZ activity in embryos was detected with the Skt^Ex5lacZ^ but not the Skt^Ex1IRESLacZ^ allele, the promoter/enhancer driving Skt/Etl4 expression during embryogenesis most likely resides within the 595 kb genomic region between exon 1 and exon 5. Similar to the existence of transcripts of different sizes we detected in brain and testis lysates proteins of different molecular weights. The faster migrating protein species of approximately 150 kDa correlates well with the SKT protein detected in lysates of intervertebral discs by Semba et al. [20]. The protein with higher molecular weight might be encoded by some other transcript or generated by posttranslational modifications.

Most of the published expression domains of Skt/Etl4 were determined indirectly using the lacZ reporter gene in Etl4^lacZ^ and Skt^Gt^ mice [19, 20, 24] or with a Skt^Cre^ allele combined with a Rosa26^lacZ^ reporter line [25]. We found additional tissues expressing Skt/Etl4 that were not described so far, for instance the epithelium of the lung and the cochlea by detecting Skt/Etl4 mRNA by in situ hybridization. Given the complex regulation and splicing patterns it appears possible that some of the endogenous expression domains were not detected due to the intronic insertion sites of the reporter constructs. Analysis of the reporter gene activity in Skt^Ex5lacZ^ embryos showed specific expression within the notochord and other tissues consistent with lacZ activity in Etl4^lacZ^ and Skt^Gt^ mice [19, 20]. However, a more thorough comparison of notochord staining of the Etl4^lacZ^ and Skt^Ex5lacZ^ alleles showed that lacZ expression along the entire length of the notochord in Etl4^lacZ^ embryo was not recapitulated in Skt^Ex5lacZ^ embryos: here the staining showed a gradient of expression with the strongest staining in the tail area. Since this notochord expression pattern was also detected in WISH experiments with the exon 1-5 probe it presumably reflects the actual endogenous gene activity and might explain the regionally tail restricted IVD phenotype in Skt/Etl4 mutants.

To study the function(s) of the brain/testis-specific and the embryonic transcripts we introduced a lacZ gene with a stop cassette (triple poly (A)), which was previously shown to effectively prevent transcriptional read-through [23], into exon 1 and 5, respectively. Insertion into exon 1 did not cause any obvious phenotype, indicating that the transcript (s) required for Skt/Etl4 function in the notochord was still functional, presumably due to a transcriptional start site downstream of exon 1. Likewise the lacZ insertion into exon 5 did not cause any obvious phenotype, despite lacZ expression in embryonic tissues similar to endogenous expression. It turned out that the integration of the transcriptional stop cassette did not eliminate Skt/Etl4 expression, but was removed from a read-through primary transcript by splicing around the lacZ cassette, indicating that the triple poly (A) signal was not sufficient to terminate transcription in this context.

Since the lacZ insertions into exon 1 or 5 did not phenocopy the Skt^Gt^ or Etl4^lacZ^ phenotype we deleted genomic DNA containing exon sequences encoding nearly 90 % of the protein deduced from the longest known open reading frame. To achieve this we deleted by site-specific inter- and intra-chromosomal recombination events using the Cre/loxP recombinase system [26–28] a total of around 870 kb of genomic DNA. The efficiency for creating genomic deletions in vivo with the TAMERE strategy decreases with size. It was successful in males for trans-located loxP sites separated by 150 kb [21, 29], but failed for loxP sites with a trans distance of 3,9 Mb [30]. We obtained a genomic deletion of 595 kb with TAMERE using females that express Cre recombinase in oocytes [ZP3::Cre 32] in one offspring within the first two litters. However, due to the small numbers we can not draw firm conclusions concerning the trans-chromosomal recombination efficiency in female mice. Excision of the residual 273 kb genomic DNA between two cis-located loxP sites that occurred in all analysed offspring (n = 26) demonstrating that an intrachromosomal deletion of this size can efficiently be obtained in the female germ line.

Our Skt/Etl4 deletion represents a bona fide null allele whose phenotype is undistinguishable from Etl4^lacZ^ and Skt^Gt^ mice. All our mice that were homozygous for the deletion and were histologically analysed (*n* = 9) showed defects in the caudal vertebral column. In contrast, in Etl4^lacZ^ and Skt^Gt^ mutants only one third or half of the mutants had obvious defects [19, 20]. Since these mice were analysed on a C57BL/6 background, whereas our mice are on a mixed CD1/129Sv background, genetic background differences might contribute to the different penetrance of the phenotype. Thus, we cannot distinguish at present whether the complete penetrance of the phenotype in our deletion mutant reflects that Etl4^lacZ^ and Skt^Gt^ represent hypomorphic alleles or is due to genetic background differences.

In Skt^Gt^ as well in Etl4^lacZ^ mutants no kidney abnormalities or impaired fertility were observed [19, 25]. We analysed selected organs of mutants highly expressing endogenous Skt/Etl4 histologically and found only abnormalities within the IVDs solely in the caudal region of the vertebral column. We cannot exclude that there are subtle defects or that other tissues that we did not analyse are affected in our deletion mutants. However, at present it appears that in almost all expression domains the loss of Skt/Etl4 function can be compensated. The specific expression domain in the NP of the IVDs and strong association of SKT polymorphisms with Lumbar disc herniation (LDH) or Disc degeneration (DD) in Finish and Japanese populations [31, 32] makes SKT a good candidate for a LDH or DD susceptibility gene in humans. These studies did not mention other health problems in these patients, supporting that Skt/Etl4 function is only required for IVD formation or stability. However, it is still unclear how the LDH associated and intronic SNPs influence SKT/ETL4 function in the patients [31].

The region on chromosome 2 that we deleted contains 11 gene predictions including small nucleolar RNAs (snoRNAs), small nuclear RNA (snRNAs), pseudogenes, protein coding genes and unclassified non-coding RNA genes (Gm25859, Gm13361, Gm13363, Gm13360, Gm13328, Gm34260, Gm23970, Gm17171, Gm13335, Gm27446, Gm13362) as well as two unclassified genes represented by cDNA clones (Gm16495, 8030447M02Rik) (http://www.informatics.jax.org). To the best of our knowledge no functional data for these gene predictions are available and is not clear whether these annotations represent true functional genes. However, as our mice are largely normal none of these annotated sequences can be essential for developmental processes, viability and fertility.

## Conclusion

Inhibition of gene expression via integration of a transcriptional stop signal (triple poly (A)) can be inefficient for gene loci with complex regulation. For genome modification inter- and intrachromosomal recombination in the female germ line can be used to delete large genomic regions. The generation of a bona fide null allele of the Skt/Etl4 gene with this technique demonstrates that this gene as well as other annotated sequences in this region are not essential for viability and fertility of mice, but Skt/Etl4 is only required for caudal intervertebral disc development. These analyses also indicate that both Etl4^lacZ^ and Skt^Gt^ abolish Skt/Etl4 function.

## Materials and methods

### Ethics statement

All animal experiments were performed according to the German rules and regulations (Tierschutzgesetz) and approved by the ethics committee of Lower Saxony for care and use of laboratory animals LAVES (Niedersächsisches Landesamt für Verbraucherschutz und Lebensmittelsicherheit; AZ 33-42502-02/543). Mice were housed in the central animal facility of Hannover Medical School (ZTL) and were maintained as approved by the responsible Veterinary Officer of the City of Hannover. Animal welfare was supervised and approved by the Institutional Animal Welfare Officer (Tierschutzbeauftragter).

### Mouse housing and husbandry conditions

Mice were bred and maintained under routine husbandry procedures following standards published by the Society for Laboratory Animal Science (SOLAS) and the EU directive 2010 63 EU at a temperature of 21 °C, relative humidity of approximately 50 %, and artificial light from 06:00–18:00 h. Mice were kept in wire-topped type IIL Makrolon cages (Techniplast, Techniplast-Deutschland GmbH, Hohenpeienberg, Germany) on sterilised softwood granulate bedding (Lignocel, Altromin; Lage, Germany) and received autoclaved commercial pellet diet (Altromin1314) (protein 22, fat 5, raw fibre 4.5, ash 7 %, utilizing energy 3.1 kcal/g) and water ad libitum. Microbiological status was measured at least every six months according to the recommendations for the health monitoring of rodent colonies in breeding and experimental units.

### Shotgun cloning and sequencing

Shotgun libraries of BAC DNA with average insert sizes of 1.5 kb and 3.5 kb were generated and sequenced as described [16].

### Northern blot analysis

Commercially available Northern blots (Mouse Embryo MTN Blot (#7763-1, Clontech), Message Map Northern Blot organs adult (#776900, Stratagene)) were hybridized with probes radioactively labelled with [α-^32^P] dCTP using the Prime-it II Random Primer Labelling kit according to the manufacture’s instructions. Blots were hybridized in ULTRAhyb® hybridization solution (Ambion) for one hour without probe, then overnight with probe at 42 °C. Unspecific probe was removed by washing with NorthernMax® Low Stringency Wash buffer (Ambion) twice for five minutes and once with NorthernMax® High Stringency Wash buffer (Ambion) for 15 min at 42 °C.

### Skt/Etl4 specific exon probes

Skt/Etl4 specific fragments that were used as probes for whole-mount in situ hybridization and Southern and Northern blot experiments were amplified by PCR and sub-cloned into pGEM®-T Easy vector (Promega). The following primer pairs for amplification from cDNA clones were used: Exon 1–3 (AAGGTAGCGGAGGCTCAAG, CCCAGTATTTCCATCCCATAG), Exon 5–8 (AGCCATCATGGGCCACC, GCATGTGAAGGATCCTTGTTG), Exon 9–13 (CCCTGATAGCCATTTGCCTACC, TTTGACAATCCGCTCTTCTTCG), Exon 15–18 (GAACAGGGCAGTGTCCATTGAG, TCTTCTTCTTCCTCCTCCTCCTTG).

### Whole-mount and section in situ hybridization

Whole-mount and section in situ hybridization of embryos was performed with digoxygenin-labelled antisense riboprobes as described [33, 34]. Documentation was done with the Leica M420 microscope with Apozoom 1:6 and the Photograb-300Z version 2.0 software. In addition to Skt/Etl4 specific probes riboprobes specific for T [35], and Shh [3] were used.

### Skeletal preparations of newborns

Newborn mice were prepared, stained and documented as described previously [36].

### Cloning of targeting constructs

Cloning of the Skt^Ex1IRESlacZ^ targeting construct: The genomic region including and surrounding Exon 1 was amplified from a mouse genomic 129Svlmj PAC-clone (RZPD PAC-Library 711; clone 109.9.161) in three steps with primer pairs CCAACTCAGGTTCTCGG, GGAGCACTTGTCCCCTTAG and ATGAGTATGAGCACATCGG, GAGATTGGGTGACTTACGG and GAGAGAGAGAGAGACTGGTCC, TCAAGGTCAGCCTGGTAG. The three fragments were linked by AccI and StuI sites, respectively. The IRESlacZ-reporter gene with a triple poly (A) signal followed by a floxed PGK-Neo polyA- cassette was introduced into the SacII site of exon 1.

Cloning of the Skt ^Ex5lacZ^ targeting construct: the 5’flank was amplified from genomic DNA with primer pair CTAATGGAGTGGTGGATGAGCG, TAACAAGAAAGGTCAGGAGCCG. The 3’flank region was amplified in two steps with primer pairs GACCAGGTAGGAACACACTATCGG, CACAATCTATTTTTAGCCGCTTTAAT and AGTGTGTAGTCCTGGAGGGC, CAAAGTATGAATGGGGGCGG. The two fragments were combined using a BstZ17I site. A lacZ-reporter gene with a triple poly (A) signal was introduced between the *Apa*I site of exon 5 and the StuI site of intron 5 into a PCR fragment obtained from genomic DNA with primers CCATCAAAACATACCCACG and TCAGATTTCAACTCAGGTCG, which leads to a 695 bp deletion of the sickle tail locus. The lacZ containing exon 5 together with the 3’flank and the 5’flank were combined and a floxed PGK-Neo cassette was inserted into a SalI site downstream of the triple poly (A) signal. In addition a DT cassette was integrated upstream of the 5’flank into the cloning vector. Cloning of the Skt ^Ex21GFP^ targeting construct: for amplification of the flanks from genomic DNA the following primer pairs were used: 5’flank primer CTGTGGTTGATACTGACTTCG, GTTGTTTATGGAAGGCGAC; 3’flank primer ACATTCCTCTCCCAACTCG, TCACTCTTTCTCAGCGTCC. The C-terminal GFP tag was fused in frame with the Skt/Etl4 cDNA using a PCR generated BglII site instead of the Skt/Etl4 stop codon. Combination of the 5’flank and 3’flank containing exon 21 with GFP was performed using a genomic SalI site. Into a genomic StuI site located in intron 20 a floxed PGK-Neo Cassette was introduced for ES cell selection. Additionally a DT cassette was integrated into the vector backbone using EcoRV and SacII sites.

### Generation of ES cells

ES cell lines homozygous for Skt ^ΔEx1-5^ were obtained from d 4.5 blastocysts collected from matings of homozygous Skt ^ΔEx1-5^ mice on a mixed CD1/129Sv/ImJ genetic background as described [37].

### Generation and genotyping of mutant mice

Positive ES cell clones that were electroporated with Skt^Ex1IRESlacZ^ or Skt^Ex5lacZ^ targeting constructs, were verified by Southern blot analysis and used for chimera production. For removal of the floxed PGKNeo cassette mice were crossed to ZP3::Cre mice [38]. Genomic DNA isolated from tails was used for genotyping using the following PCR primer pairs (see also Fig. 4): PCR 1 (GCTCCCAACTCTACCCAGAC, CCCTCACATTGCCAAAAGACG, 679 bp), PCR 2 (TCAGCCATACCACATTTGTAGAG, CTGGGGAGACGACTTTCAAG, 280 bp), PCR 3 (TCGTTAGCAACTGCCACAACC, TCGCCGCACATCTGAACTTC, 964 bp), PCR 4 (AACCTCCCACACCTCCCCTG, GCAAGACTGGTCCCCAAAATAAG, 624 bp), PCR 5 (TGGAAGTTCAAGCAAAGCC, TTGTGCCCCAGGATGTTG, 492 bp), PCR 6 (AACCTCCCACACCTCCCCTG, ACAGCCCTTCTGAGCATCATTTAG, 404 bp). For Southern blot analysis a 1,9 kb ApaI/SacI fragment of the lacZ coding sequence was used as a probe. An 889 bp Skt/Etl4 specific probe 3’to exon 21 (3’Ex21) was generated by PCR with primers TGAGTGGCATCATAATGGTGTGG and AAATACAGAGAGGAGGACAGGCGG.

### RT-PCR

RT-PCR was performed with poly (A^+^) RNA and the Thermoscript RT-PCR system (Invitrogen) according to the manufacturer’s instructions with primer pairs: forward-primer exon 1 (AAGGTAGCGGAGGCTCAAG), backward primer exon 3 (CCCAGTATTTCCATCCCATAG) forward primer 4 (CCAGAAATGTGAGCCGAAC), backward-primer exon 5 (TCCATTAGAAAGGCGTTCCC), backward-primer lacZ (TCGCCGCACATCTGAACTTC), backward-primer exon 7 (TGTCTGTGCTTGTGACTTCATTCG), forward-primer exon 20 (CTTCCAAGAACAGACCCG), backward-primer exon 21 (CTTTCTTAGCACTTCCATTAGC).

### β-galactosidase (lacZ) staining of embryos

β-galactosidase activity was detected as described [39]. Briefly, embryos were fixed for 5 min in 0,1 M phosphate buffer (pH 7,4) with 0,4 % glutardialdehyde, 2 mM MgCl_2_ and 5 mM EGTA, and washed three times for 10 min in 0,1 M phosphate buffer (pH 7,4) with 2 mM MgCl_2_, 0,1 % sodium deoxycholate and 0,02 % NP-40. Staining was performed at 37 °C in 0,1 M phosphate buffer (pH 7,4) with 1 mg/ml X-Gal (5-bromo-4-chloro-3-indoyl-β-D-galactoside), 5 mM potassium ferrocyanide and 5 mM potassium ferricyanide and stopped with 4 % paraformaldehyde.

### Histological analysis

Kidney, lung, vertebral columns (sacral and caudal) and isolated inner ears of three week old mice were fixed in 4 % PFA overnight, decalcified in 0,5 M EDTA/PBS for 2 days (inner ears) or 2 weeks (vertebrae) respectively, dehydrated, paraffin wax embedded sectioned to 10 μm and stained with haematoxylin and eosin according to standard procedures.

### SKT/ETL4-specific antibodies and Western blot analysis

SKT/ETL4 specific antibodies (anti-NGS) were generated in rabbit (Biogenes GmbH, Berlin, Germany) against the C-terminal protein sequence NGSSSKATPSTAKETS and affinity purified as described [40]. Affinity purified anti-NGS antibodies were used for Western blot analyses diluted (1:750) in PBS containing 5 % Milk powder and 0,5 % Tween followed by an incubation with horseradish peroxidase-conjugated anti-rabbit antibody (GE Healthcare) and detection by ECL.

## Availability of supporting data

All supporting data are included in Supporting Information files.

## Additional files

Additional file 1: Figure S1. lacZ reporter gene activity in Skt^Ex1IRESlacZ^ and Skt^Ex5lacZ^ mice. (A) β-Galactosidase staining of wt (a, c) and homozygous Skt^Ex1IRESlacZ^ (b, d) adult testes and epididymides. (B) β-Galactosidase staining of wt (a, d), heterozygous (b, e) and homozygous (c, f) Skt^Ex5lacZ^ E10.5 and E11.5 embryos. White triangles in (b, c, e and f) point to lacZ expression in the caudal notochord. (TIF 8102 kb) Additional file 2: Figure S2. Insertion of triple poly (A) into exon 5 of the Skt/Etl4 does not prevent transcription of downstream exons. (A) Schematic representation of the Skt^Ex5lacZ^ allele and location of PCR primer pairs used for RT-PCR. (B) RT-PCR results with PCR primer pairs depicted in (A) with poly (A^+^) RNA isolated from E10.5 wt, heterozygous and homozygous Skt^Ex5lacZ^ embryos. (TIF 186 kb) Additional file 3: Figure S3. Restriction maps of *Skt/Etl4* alleles. Schematic representation of the relevant regions of various Skt/Elt4 wt and mutant alleles with location of probes, restriction sites and expected restriction fragments detected by Southern blot hybridizations shown in Fig. 4e. An EcoRV digest of wt DNA results in a 15 kb fragment detected by the 3’Exon21 probe (A and Fig. 4e c), which shifts down to 11 kb in the Skt^ΔEx1-20^ allele (B and Fig. 4e c). A lacZ probe detects in Skt^Ex1IRESlacZ^ DNA digested with PstI or NcoI a 5 kb or 7.1 kb fragment (C and Fig. 4e a and b), in Skt^ΔEx1-5^ DNA a 7.7 kb or 7.9 kb fragment (D and Fig. 4e a and b), and in Skt^Ex5lacZ^ DNA a 12.6 kb or 12 kb fragment (E and Fig. 4e a and b). (TIF 413 kb)

## Abbreviations

AER: apical ectodermal ridge
AF: annulus fibrosus
BAC: bacterial artificial chromosome
DT: diphtheria toxin A
EDTA: ethylene demine tetraacetic acid
EP: endplate
ES cell: embryonic stem cell
Etl4: enhancer trap locus 4
Etl4^lacZ^: Etl4 mutagenesis line
FOXA2: forkhead box protein A2
GFP: green fluorescent protein
HE: Haematoxylin-Eosin
IRES: internal ribosome entry site
IVD: intervertebral disc
lacZ: β-Galactosidase gene
loxP: LoxP sequence
NCBI: National Centre for Biotechnology Information
Neo: neomycin
Noto: notochord homeobox
NP: nucleus pulposus
PAC: P1 artificial chromosome
PBS: phosphate buffered saline
PGK: phosphoglycerate kinase 1 promoter
poly (A): polyadenylation signal
SDS-PAGE: sodium dodecyl sulphate polyacrylamide gel electrophoresis
Shh: sonic hedgehog
SISH: section in situ hybridization
Skt: sickle tail locus
Skt^GT^: Skt mutagenesis line
snoRNAs: small nucleolar RNAs
snRNAs: small nuclear RNA
Sox5: SRY-Box 5
Sox6: SRY-box6
T: brachyury
TAMER: targeted meiotic recombination
TEAD1: TEA domain family member 1
TEAD2: TEA domain family member 2
WISH: whole mount in situ hybridization
