# Supplementary figures and images for: Generation of an 870 kb deletion encompassing the Skt/Etl4 locus by combination of inter- and intra-chromosomal recombination
Source: BMC Genet. 2015 Dec 18;16:143. doi: 10.1186/s12863-015-0302-0 (PMC4683868; doi:10.1186/s12863-015-0302-0)

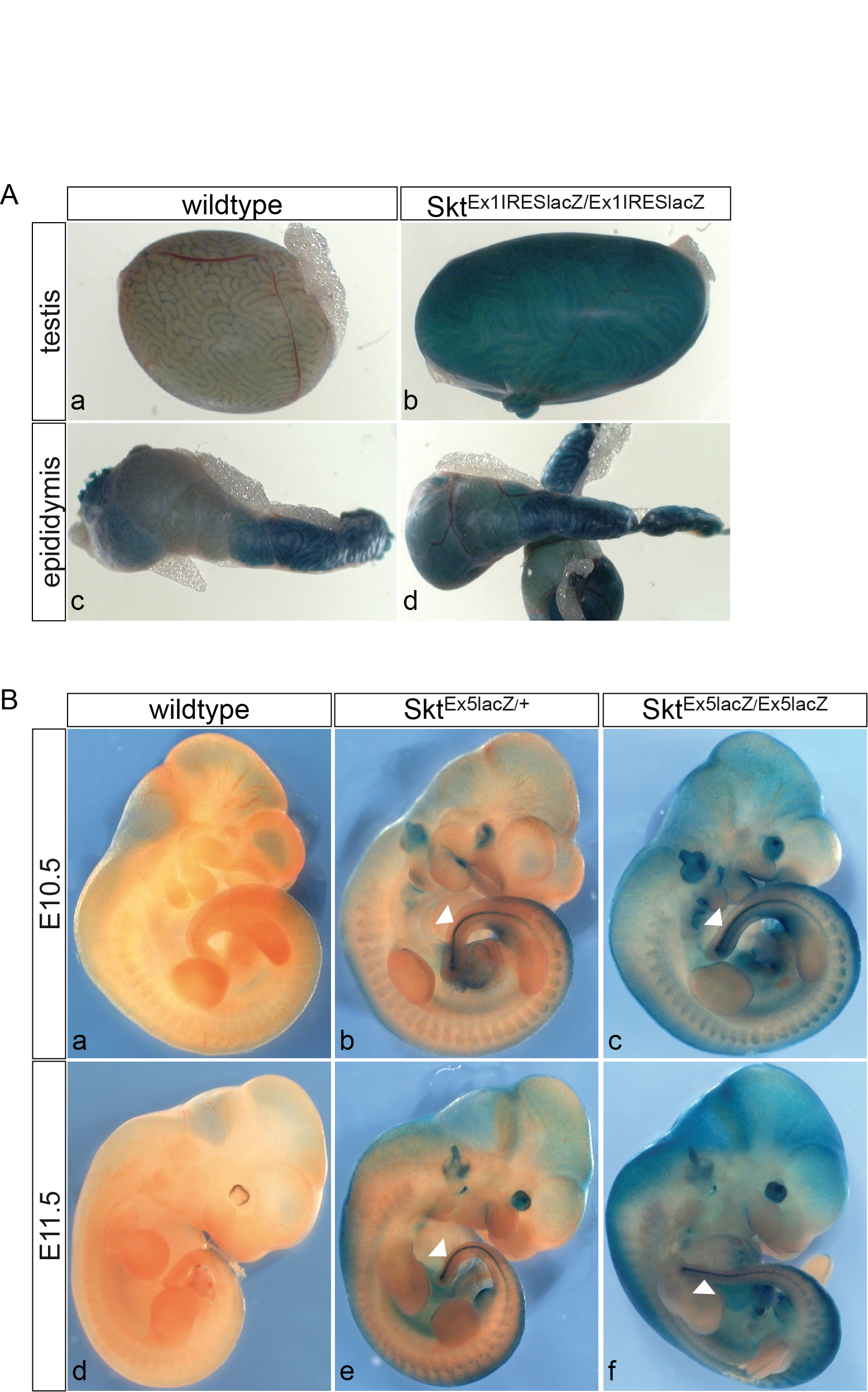

Supplement: Additional file 1: Figure S1. — lacZ reporter gene activity in SktEx1IRESlacZ and SktEx5lacZ mice. (A) β-Galactosidase staining of wt (a, c) and homozygous SktEx1IRESlacZ (b, d) adult testes and epididymides. (B) β-Galactosidase staining of wt (a, d), heterozygous (b, e) and homozygous (c, f) SktEx5lacZ E10.5 and E11.5 embryos. White triangles in (b, c, e and f) point to lacZ expression in the caudal notochord. (TIF 8102 kb) [file 12863_2015_302_MOESM1_ESM.tif]

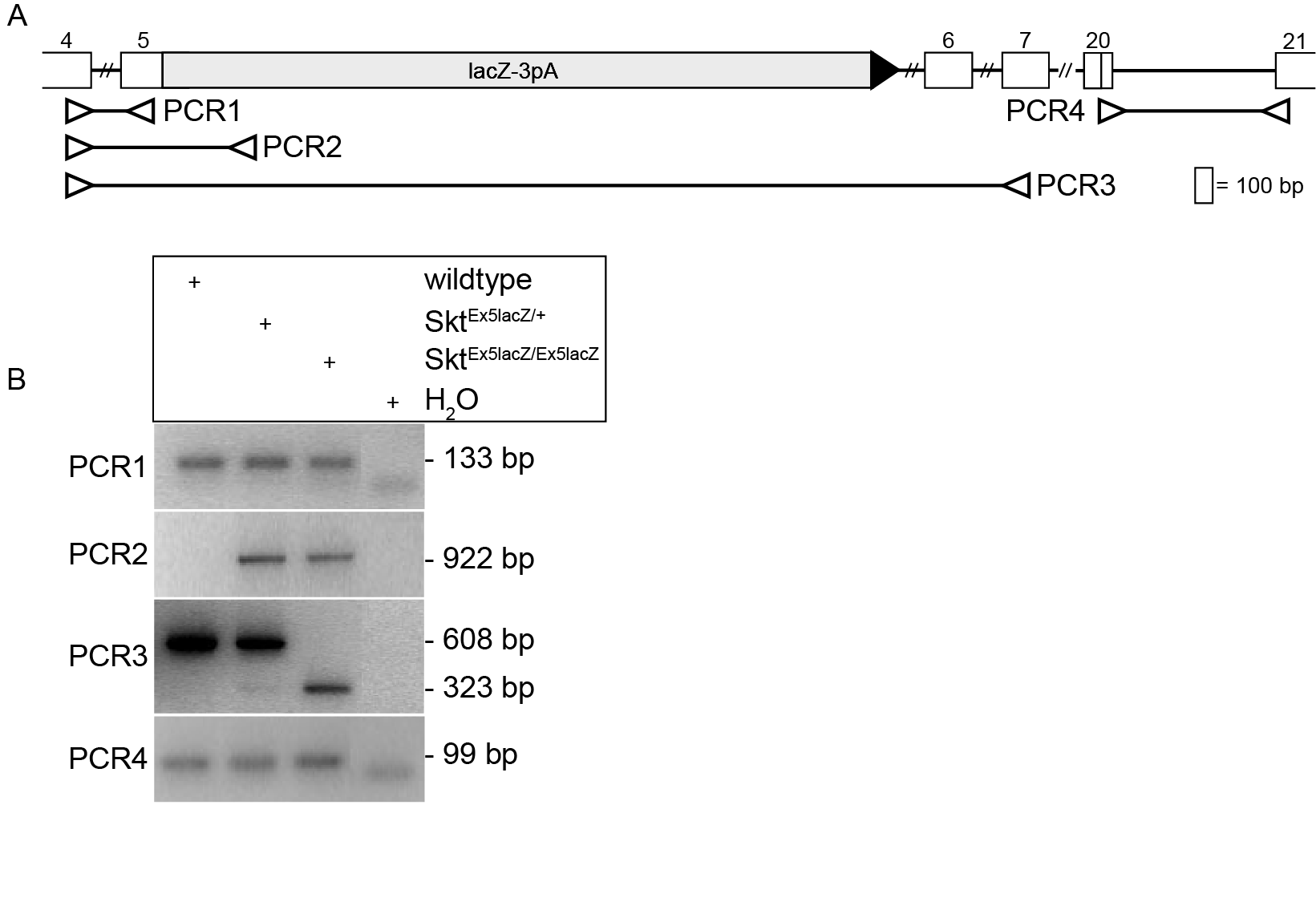

Supplement: Additional file 2: Figure S2. — Insertion of triple poly (A) into exon 5 of the Skt/Etl4 does not prevent transcription of downstream exons. (A) Schematic representation of the SktEx5lacZ allele and location of PCR primer pairs used for RT-PCR. (B) RT-PCR results with PCR primer pairs depicted in (A) with poly (A+) RNA isolated from E10.5 wt, heterozygous and homozygous SktEx5lacZ embryos. (TIF 186 kb) [file 12863_2015_302_MOESM2_ESM.tif]

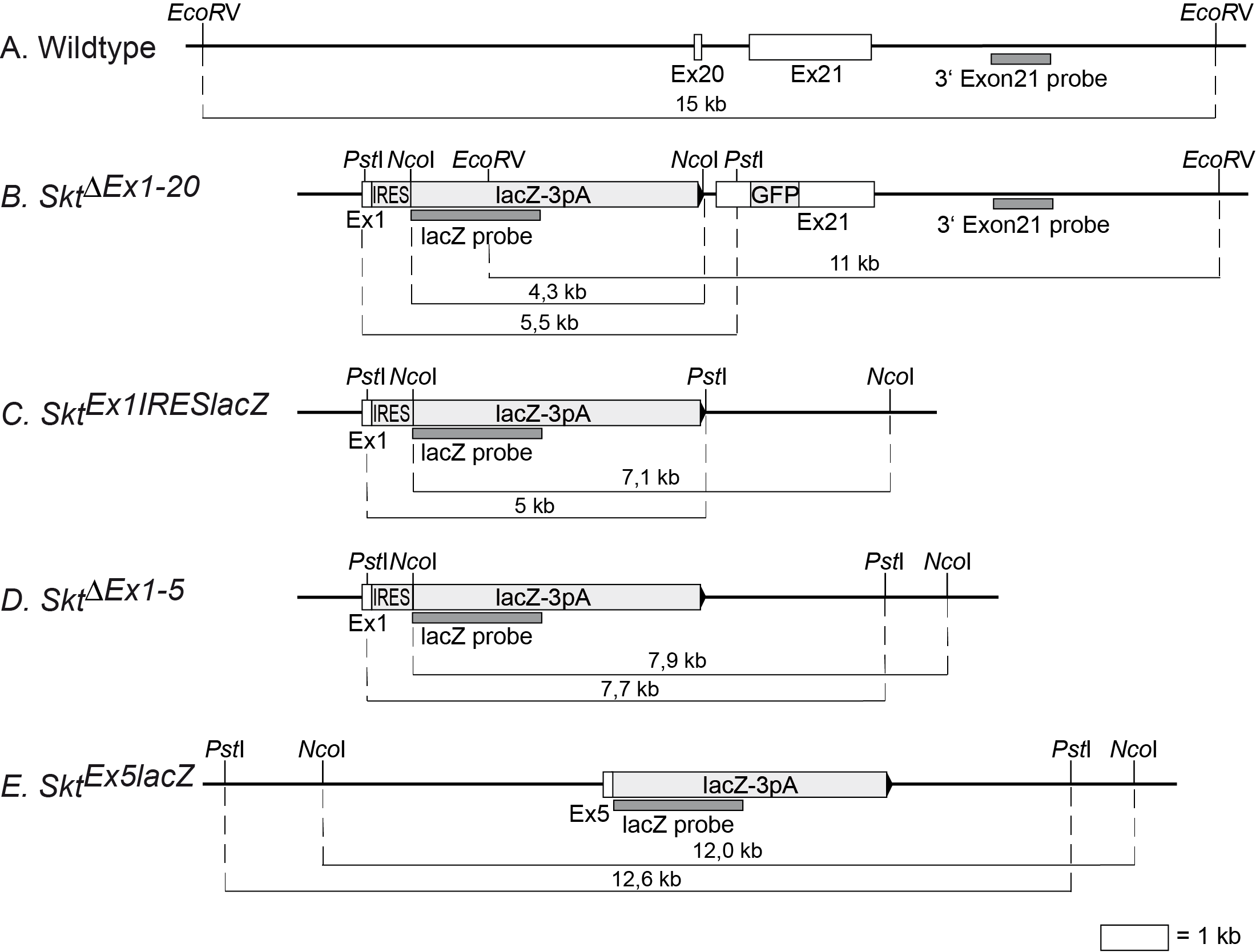

Supplement: Additional file 3: Figure S3. — Restriction maps of Skt/Etl4 alleles. Schematic representation of the relevant regions of various Skt/Elt4 wt and mutant alleles with location of probes, restriction sites and expected restriction fragments detected by Southern blot hybridizations shown in Fig. 4e. An EcoRV digest of wt DNA results in a 15 kb fragment detected by the 3’Exon21 probe (A and Fig. 4e c), which shifts down to 11 kb in the SktΔEx1-20 allele (B and Fig. 4e c). A lacZ probe detects in SktEx1IRESlacZ DNA digested with PstI or NcoI a 5 kb or 7.1 kb fragment (C and Fig. 4e a and b), in SktΔEx1-5 DNA a 7.7 kb or 7.9 kb fragment (D and Fig. 4e a and b), and in SktEx5lacZ DNA a 12.6 kb or 12 kb fragment (E and Fig. 4e a and b). (TIF 413 kb) [file 12863_2015_302_MOESM3_ESM.tif]
